# Characterization of Plasmodium infections among inhabitants of rural areas in Gabon

**DOI:** 10.1038/s41598-019-46194-9

**Published:** 2019-07-05

**Authors:** Tamirat Gebru Woldearegai, Albert Lalremruata, The Trong Nguyen, Markus Gmeiner, Luzia Veletzky, Gildas B. Tazemda-Kuitsouc, Pierre Blaise Matsiegui, Benjamin Mordmüller, Jana Held

**Affiliations:** 1 Institut für Tropenmedizin, Eberhard Karls Universität Tübingen, Tübingen, Germany; 2 German Centre for Infection Research, partner site Tübingen, Tübingen, Germany; 3 Centre de Recherches Médicales de Lambaréné (CERMEL), Lambaréné, Gabon; 4 Vietnamese – German Center of Excellence in Medical Research, Hanoi, Vietnam; 5 Centre de Recherches Médicales de la Ngounié, Fougamou, Gabon; 6 Department of Tropical Medicine, Bernhard Nocht Institute for Tropical Medicine & I. Dep. of Medicine, University Medical Center Hamburg-Eppendorf, Hamburg, Germany

**Keywords:** Microbiology, Haplotypes

## Abstract

*Plasmodium* infections in endemic areas are often asymptomatic, can be caused by different species and contribute significantly to transmission. We performed a cross-sectional study in February/March 2016 including 840 individuals ≥ 1 year living in rural Gabon (Ngounié and Moyen-Ogooué). *Plasmodium* parasitemia was measured by high-sensitive, real-time quantitative PCR. In a randomly chosen subset of *P*. *falciparum* infections, gametocyte carriage and prevalence of chloroquine-resistant genotypes were analysed. 618/834 (74%) individuals were positive for *Plasmodium* 18S-rRNA gene amplification, of these 553 (66.3%) carried *P*. *falciparum*, 193 (23%) *P*. *malariae*, 74 (8.9%) *P*. *ovale curtisi* and 38 (4.6%) *P*.*ovale wallikeri*. Non-falciparum infections mostly presented as mixed infections. *P*. *malariae* monoinfected individuals were significantly older (median age: 60 years) than coinfected (20 years) or *P*. *falciparum* monoinfected individuals (23 years). *P*. *falciparum* gametocyte carriage was confirmed in 109/223 (48.9%) individuals, prevalence of chloroquine-resistant genotypes was high (298/336, 89%), including four infections with a new SVMNK genotype. In rural Gabon, *Plasmodium* infections with all endemic species are frequent, emphasizing that malaria control efforts shall cover asymptomatic infections also including non-falciparum infections when aiming for eradication.

## Introduction

Due to huge efforts in elimination and eradication of malaria the numbers of malaria cases and deaths reduced substantially according to WHO in recent years^1^. Despite this, between 2014 and 2016 there was a considerable increase in number of malaria cases, showing that it needs continuous efforts to have sustainable success. To reach the ambitious aim of eradication, control measures have to remain constantly high also including asymptomatic and submicroscopic carriers to eliminate this constant reservoir. Malaria control efforts should additionally include remote areas, where maintaining health care is difficult. This investigation analyzed by molecular methods the silent reservoir of malaria parasites in a rural area of Gabon, Central Africa.

We did a cross-sectional survey to investigate and further characterize the different *Plasmodium* species prevalent in a rural population in the area of Fougamou, Gabon. *P*. *malariae* and *P*. *ovale* infections (*P*. *ovale wallikeri* and *P*. *ovale curtisi*) are commonly underreported as parasitemia is often low and severe disease is uncommon^2,3^. However, infections by these species can be chronic and contribute significantly to morbidity by e.g. causing anemia^4^. A previous study in the same area on symptomatic patients with malaria caused by non-*falciparum/*mixed species infections revealed an unexpected complexity of infections when analysis was done by deep sequencing technology^5^. Within the current study, the submicroscopic and microscopic prevalence of the different *Plasmodium* species in the resident population was investigated before we analysed in more depth the most virulent and abundant parasite *P*. *falciparum* by evaluating prevalence of gametocytes and the presence of chloroquine resistant genotypes.

Recently recognized as an important malaria transmission reservoir, asymptomatic infections and submicroscopic gametocytemia are major hurdles for malaria elimination^6^. Control and elimination strategies of malaria therefore need to employ highly sensitive techniques to identify such reservoirs. One approach is to assess *P*. *falciparum* gametocyte carriage by quantitative real-time PCR (qPCR) based on the marker Pfs25. In addition, molecular markers for drug-resistance and other polymorphisms can be analyzed to inform the best intervention strategies. E.g., in many African countries chloroquine sensitivity of *P*. *falciparum* returned after change of treatment policy and subsequent withdrawal of chloroquine from the market^7^, but this was not yet observed in Gabon^8,9^ despite discontinuation of chloroquine for malaria treatment in Gabon since 2003^10^.

With this study, we aimed at characterizing the whole *Plasmodium* population infecting humans in Fougamou, Gabon. This is done by assessing prevalence of different *Plasmodium* species, *P*. *falciparum* gametocyte carriage and prevalence of *P*. *falciparum* chloroquine resistance by qPCR in a cross-sectional survey.

## Results

**Figure 1:**
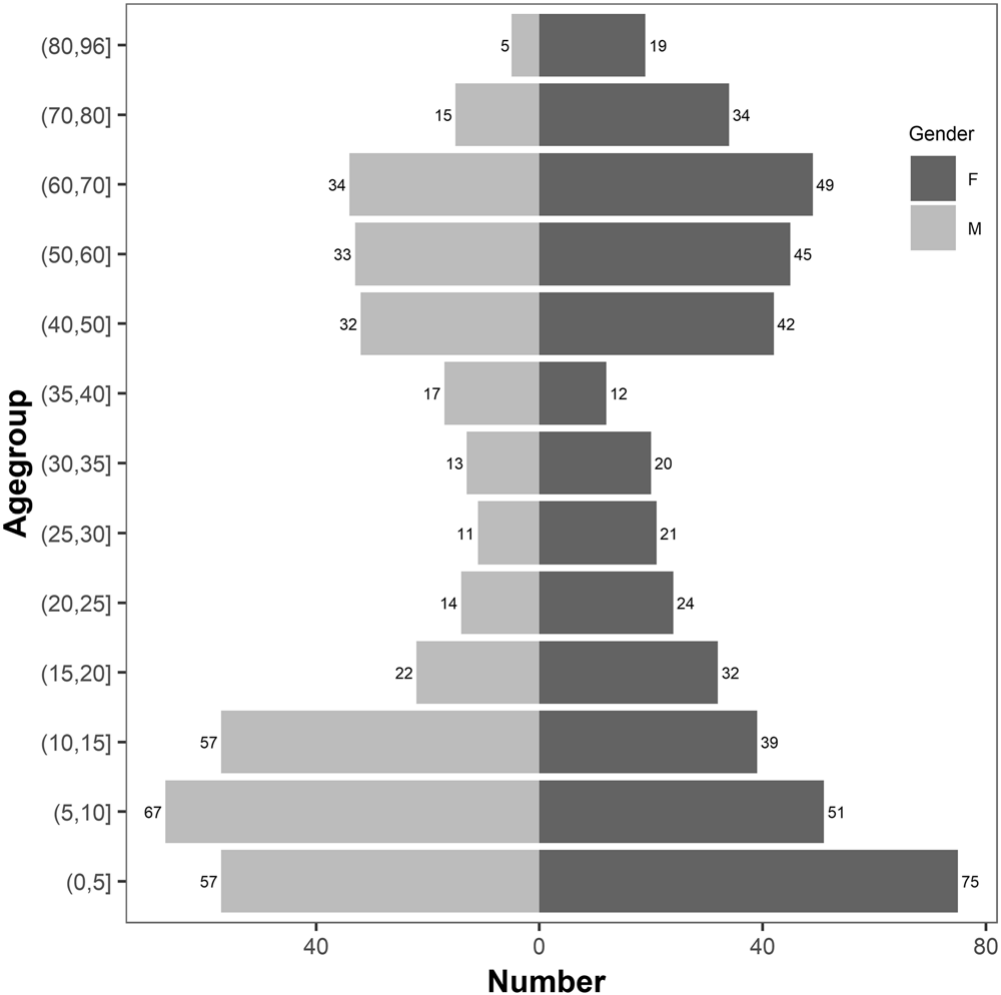
Demographic characteristics of study population.

We included 840 individuals and obtained blood of 834 persons, description of the study population is given in Fig. 1. 311/834 (37%) individuals were positive for *Plasmodium* parasites by Giemsa stained thick blood smear, 72 (8.6%) of them were considered as being mixed or non-*falciparum* infections. Microscopic results have been published before^11^. Body temperature was recorded for 771 individuals, mean temperature was 37.1 °C; 24 individuals presented with a body temperature ≥ 38 °C, 14 of those 24 had a parasitemia that could be detected by microscopy.

### Microscopic reading versus PCR

PCR and microscopy reading results were well correlated (r = 0.7403, p < 0.0001). All PCR samples with a Cq < 17.7 (n = 85) were microscopically positive, and all samples with a Cq > 29 were microscopically negative (n = 76), hence below the limit of detection for microscopy. 214/217 (98.6%) PCR negative samples were also read negative by microscopy, three were read false positive with a low parasitemia (below 25 parasites/µl) confirming the accuracy of our microscopy results. Non-falciparum species were not further distinguished by microscopy, but reported as such, so that only differentiation of *P*. *falciparum* versus non-falciparum species was performed.

### PCR results for *Plasmodium* species

**Figure 2:**
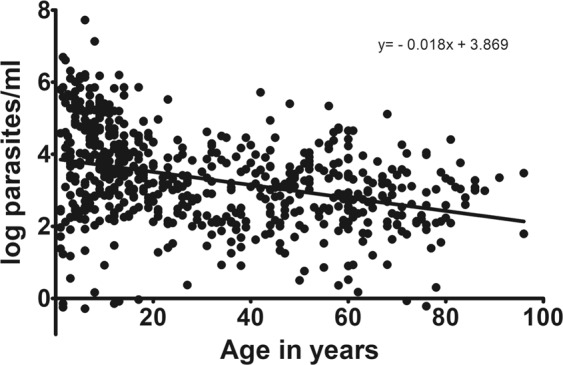
Age versus Plasmodium parasitemia. Age of participants versus log number of parasites (pan-Plasmodium RNA and DNA) per ml of blood (derived of qPCR data). Results show that the parasitemia declines with age. Parasitemia has been extrapolated from qPCR data with the help of a standard curve.

**Figure 3:**
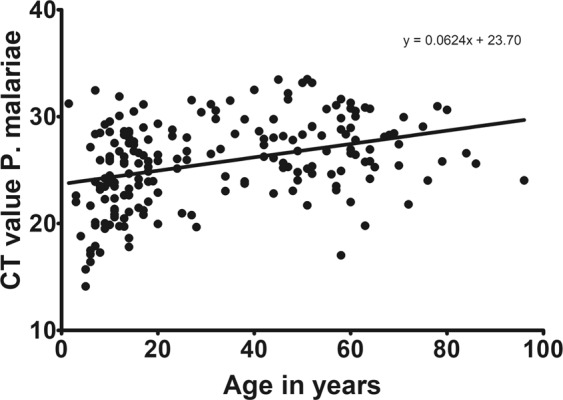
Age versus *P*. *malariae* parasitemia. Age of participants versus Cq values for *P*. *malariae*. Results show that also for *P*. *malariae* there is a decrease of parasitemia (indicated by raising Cq) with age.

Further analysis by qPCR revealed an unexpected complexity of infections and high prevalence of non-falciparum coinfections. In total 618/834 (74%) individuals were positive for 18 S plasmodial DNA, of these 553 (66.3%) were positive for *P*. *falciparum*, 193 (23%) for *P*. *malariae*, 74 (8.9%) for *P*. *ovale curtisi* and 38 (4.6%) for *P*. *ovale wallikeri*. Parasitemia of individuals decreased with age (p < 0.0001 Fig. 2). Number of parasites per microliter was calculated with the help of a standard curve. The parasitemia of *P*. *malariae* positive individuals also decreased with age (p < 0.0001, Fig. 3). However, these data should not be over-interpreted as species-specific PCR was done after preamplification.

Most non-*falciparum* infections presented as mixed infections with *P*. *falciparum*. In five individuals, all four prevalent species were found at the same time. The different combinations of infections can be seen in Table 1. *P*. *malariae* was the most abundant non-falciparum parasite, 90.6% (175/193) infections presented as coinfections with another species. We did not find any *P*. *vivax* infection in our samples.

**Table 1:** Number of mono and multiple infections of the different *Plasmodium* species.

Plasmodium species infection		
Multiplicity of infection	Number of individuals and %	
	PCR	Microscopy**
Pf monoinfection	357 (43)	239 (28.7)
Pm monoinfection	18 (2)	NA
Poc monoinfection	7 (0.8)	NA
Pow monoinfection	1 (0.1)	NA
Mixed infection (Pf + non-falciparum)	592 (71)	72 (8.6)
Pf + Pm	123 (14.7)	NA
Pf + Poc	21 (2.5)	NA
Pf + Pow	18 (2.1)	NA
Pm + Poc	1 (0.1)	NA
Pf + Pm + Poc	36 (4.3)	NA
Pf + Pm + Pow	10 (1.1)	NA
Pf + Poc + Pow	4 (0.5)	NA
Pf + Pm + Poc + Pow	5 (0.6)	NA
Pan-Plasmodium positive but species negative	17 (2)	NA
Total positive	618 (74)	311 (37.3)
Total analysed	834*	834*

NA: not applicable (Microscopic result was limited to separating *P*. *falciparum* from non-*falciparum*, therefore each species was not determined by microscopy).

*‘Total analysed’ (N = 834) is the denominator for calculating ‘percentage of individuals infected’.

**Microscopy data already published^11^.

### Individuals with a monoinfection of a non-falciparum species

**Figure 4:**
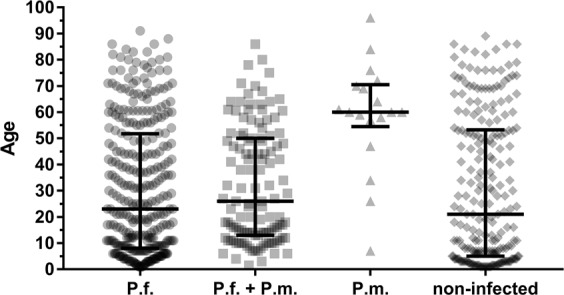
Age of *P*. *malariae* monoinfected individuals. Median age and interquartile range of *P*. *falciparum* monoinfected individuals (P.f.), versus age of coinfected individuals with *P*. *falciparum* + *P*. *malariae* (P.f. + P.m.) versus *P*. *malariae* monoinfected (P.m.) versus non-infected individuals. *P*. *malariae* monoinfected individuals are significantly older than Pf + Pm coinfected individuals (p < 0.0001).

There were only 26/834 individuals who had a monoinfection of a *Plasmodium* species that was non-falciparum. Median age of the *P*. *malariae* monoinfected individuals (n = 18) was 60 years (interquartile range 57.25–69.75 years) and therefore significantly older than for the individuals having a mixed infection of *P*. *malariae* with *P*. *falciparum* (n = 174, median age = 20 years, IQR: 11.75–49 years; p < 0.0001, U test, see Fig. 4). *P. falciparum* monoinfected individuals had a median age of 23 years (IQR: 8–52 years, n = 357). For the other non-falciparum infections sample size was small, for *P*. *ovale curtisi* monoinfected individuals (n = 7) the median age was 42 years (IQR: 10–60 years) versus 14 years (IQR: 9–46 years) in individuals having a mixed infection of *P*. *ovale curtisi* with *P*. *falciparum* (n = 43). Only one individual aged 8 years had a *P*. *ovale wallikeri* monoinfection. In general, a trend towards higher age in mono-infected subjects is present.

### Age of *Plasmodium* species positives

When dividing the population in age groups and looking at the pan-*Plasmodium* positive samples, one can see that in all age groups an evenly high proportion of individuals was infected (around 70%) (Table 2). Only the very young were less infected as only 13/33 (39.4%) children with 12–23 months were infected, and 10/20 (50%) of the 24–25 months old children were infected. Parasitemia was highest in the young children and declined with age (Fig. 2).

**Table 2:** Individuals positive for the different *Plasmodium* species per age group.

Age group (years)	N screened	Pan-Plasmodium n (%)	Pf n (%)	Pm n (%)	Pow n (%)	Poc n (%)
1–5	132 (100%)	74 (56.1)	70 (53)	6 (4.5)	4 (3.0)	5 (3.8)
6–10	118 (100%)	91 (77.1)	88 (74.6)	30 (25.4)	8 (6.8)	13 (11.0)
11–15	96 (100%)	80 (83.3)	78 (81.3)	34 (35.4)	11 (11.5)	20 (20.8)
16–20	54 (100%)	45 (83.3)	44 (81.5)	22 (40.7)	3 (5.6)	6 (11.1)
21–25	38 (100%)	28 (73.7)	28 (73.7)	5 1(3.2)	0	3 (7.9)
26–30	32 (100%)	24 (75)	23 (71.9))	7 (21.9)	2 (6.3)	2 (6.3)
31–40	63 (100%)	51 (81)	48 (76.2)	13 (20.6)	2 (3.2)	3 (4.8)
41–50	73 (100%)	58 (79.5)	55 (75.3)	22 (30.1)	2 (2.7)	5 (6.8)
51–60	94 (100%)	70 (74.5)	60 (63.8)	27 (28.7)	3 (3.2)	11 (11.7)
61–70	67 (100%)	49 (73.1)	41 (61.2)	17 (25.4)	0	5 (7.5)
71–80	49 (100%)	33 (67.3)	27 (55.1)	7 (14.3)	1 (2.0)	1 (2.0)
81–96	24 (100%)	15 (62.5)	12 (50.0)	3 (12.5)	0	0
Sum	840	618	574	193	36	74

Number and percentage of individuals screened and positive for the different *Plasmodium* species in the different age groups. One individual can be positive for different species. Age groups from 30 years on comprise 10 years.

When having a closer look at the non-falciparum infections, a similar pattern to the *P*. *falciparum* infection status can be seen. All age groups were similarly infected, with a peak in the adolescents and less infections in the very young.

### *P*. *falciparum* analysis

#### *P*. *falciparum* gametocyte results

**Figure 5:**
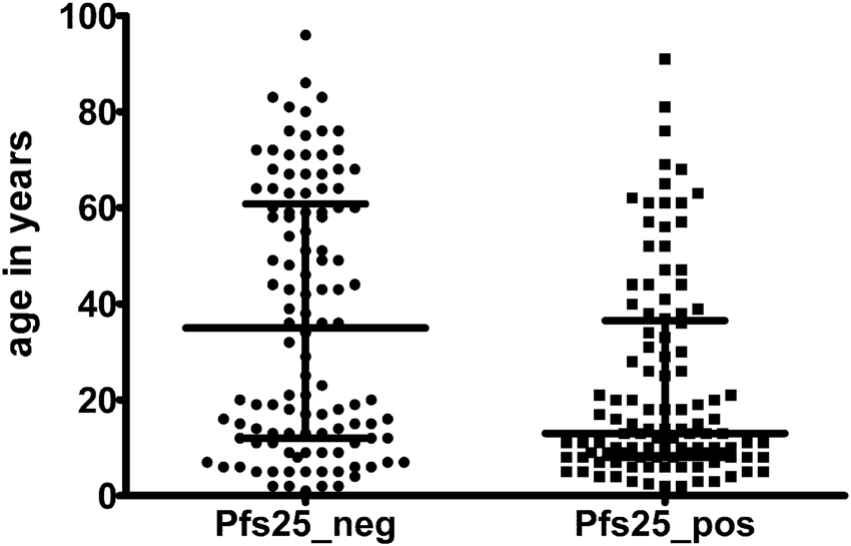
Age of gametocyte carriers. Median age and interquartile range of the subset of individuals analysed for Pfs25 positivity and negativity. Pfs25 positive individuals were significantly younger than Pfs25 negative individuals (p = 0.0002).

109 of the subset of 223 individuals were positive for Pfs25 by qPCR, of these 70 were also positive by microscopy. Age of Pfs25 positive individuals was significantly lower (median age = 13 years) than age of non-carriers (median age = 35 years), (p = 0.0002, U-test, Fig. 5). No age dependency in number of gametocytes was found.

There was a significant but weak correlation of asexual infection with gametocyte carriage (rho = 0.194, p < 0.05). Though not significant, the odds of carrying gametocytes in infected individuals was 1.5 (95% CI: 0.9, 2.5) times higher in microscopy positive than submicroscopic participants showing the influence of asexual infection on gametocyte carriage.

*P*. *malariae* coinfection neither affected gametocyte carriage rate for *P*. *falciparum* (35 *P*.*malariae* positive individuals were positive for Pfs25, while 32 were negative for Pfs25), nor gametocytemia (median Pfs25 Cq of *P*.*malariae* carriers = 30.47 and non-carriers = 30.45).

### Prevalence of chloroquine resistant *Plasmodium falciparum*

**Figure 6:**
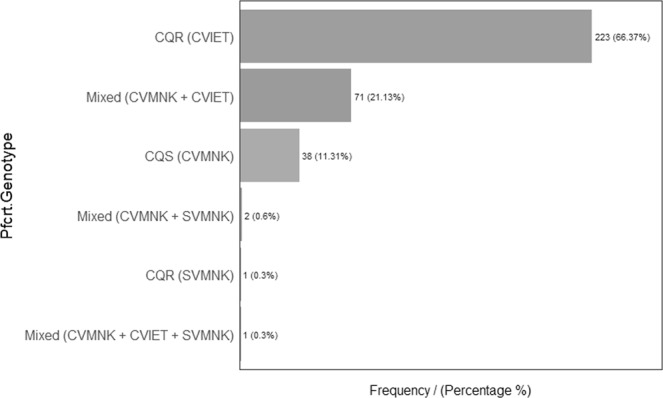
Distribution of PfCRT genotypes.

Of 336 samples screened for the three *pfcrt* haplotypes, 223 (66.4%) had CVIET haplotype only, 38 (11.3%) had CVMNK only, and 1 (0.3%) harbored a SVMNK monoinfection. The rest carried double and triple infections of wild and mutant haplotypes as; CVMNK and CVIET in 71 (21.1%), CVMNK and SVMNK in 2 (0.6%), and 1 triple infection (0.3%) as shown in Fig. 6. Overall, the prevalence of CQ resistance remained at 89% sixteen years after the withdrawal of the drug from national treatment guidelines.

## Discussion

Analysis by highly sensitive qPCR of blood samples of a rural population in Gabon revealed an unexpected breath of infection with 74% of individuals being infected with any *Plasmodium* species. Infection was evenly distributed over all age groups, with the exception that very young children were less infected maybe be due to increased care by the parents. Highest prevalence was found in children/adolescents between 5–20 years as typical for endemic areas^12,13^. There is no indication that immunity against malaria wanes in very old age in our study population. Results are in line with other studies from rural areas in Gabon showing no decline in malaria transmission in recent years^14^ but in contrast with findings from urban areas^15,16^ and results from the RTSS vaccine trial^17^ where a much lower prevalence of *Plasmodium* infections was detected.

Parasitemia was higher in very young children and was decreasing with age as expected for a population in a highly endemic area. Prevalence of non-falciparum infections was higher than expected. Nearly 50% of all infections presented as coinfections with a non-falciparum species, with *P*. *malariae* being the most prevalent. Frequent multi-species infections have been shown for certain communities harboring, low-density parasitemias of several species^3^. We could not find a single *P*. *vivax* infection in the investigated population neither as mono nor as co-infections. In fact, *P. vivax* has never been reported from Gabon. This is in line with the dogma that *P*. *vivax* is not present in Central/Western African populations as they are largely Duffy negative and *P*. *vivax* usually cannot infect Duffy negative individuals. However, our finding contradicts with recent findings reporting *P*. *vivax* presence in many regions across malaria-endemic Africa including countries bordering Gabon even though only at very low frequencies^18,19^. Reports show that *P*. *vivax* is regularly infecting Duffy-negative individuals in Madagascar^20^, and is present at low frequencies across most malaria endemic regions in Africa, infecting Duffy positive and Duffy negative individuals^21,22^. One hypothesis is that African wild living apes are infected with closely related *P*. *vivax* strains, that could in rare cases infect humans^23^. We could not see this in our samples, even though in national parks close to our sampling area wild apes are present. However, maybe the distance to this region is too big, or our sample size was too small. Further studies on *P*. *vivax* in Africa as well as in Gabon are needed to further elucidate this question. *P*. *malariae* is the second most abundant human malaria parasite after *P*. *falciparum* in many African countries^2,24,25^. Generally, quartan malaria caused by *P*. *malariae* is regarded as benign malaria even though it has been associated with nephrotic syndromes especially in young children^26^ and more long-lasting and chronic illness associated with anaemia^3^. A recent hospital-based survey studied systematically clinical symptoms due to *P*. *malariae* in Papua New Guinea discovering that even though a rare disease, children hospitalized with *P*. *malariae* also developed severe symptoms and even died^4^. The authors concluded that *P*. *malariae* is associated with anemia and a similar risk of mortality when hospitalized compared to children hospitalized with malaria caused by another *Plasmodium* (*P*. *falciparum*, *P*. *vivax*) species.

The burden of disease caused by *P*. *malariae* and *P*. *ovale*species is not well investigated. Hypnozoites of *P*. *ovale*. species infections in this area are usually not treated with primaquine/tafenoquine as glucose 6 phosphate dehydrogenase (G6PD) deficiency is prevalent in this populations, testing of G6PD-deficiency is currently not reliably possible in field settings and treatment without prior testing is prohibited^27,28^. A study in Gabon investigating the efficacy of artemether-lumefantrine for the treatment of non-falciparum and mixed species infections found good efficacy for this standard drug combination in this indication^28^. However, others found that the standard treatment for *P*. *falciparum* might not be ideal to clear non-falciparum parasites completely^2,29–31^.

Correlation of qPCR parasitemia data with age of *P*. *malariae* infected individuals indicated that immunity against *P*. *malariae* is also – as for *P*. *falciparum* – build up with age, as parasite load was higher in younger individuals than in older ones. However, these data have to be interpreted with a caveat because preamplification was done before the species-specific PCR. Monoinfections with non-falciparum species only rarely appeared and most *P*. *malariae* infections presented as coinfections with *P*. *falciparum*. Monoinfections of *P*. *malariae* (18/193) were mainly found in older individuals. We have no explanation for this finding but one hypothesis could be that even though immunity to *P*. *malariae* is built up with age similarly to *P*. *falciparum*, but this immunity with old age is less complete for *P*. *malariae* than for *P*. *falciparum* as there is less exposure due to lower parasite load/infection intensity/immune stimulation. Further studies are needed to elucidate this further.

In malaria endemic regions, asymptomatic *P*. *falciparum* infections are commonly present and reported from different transmission settings^32–34^ showing some level of dependency with age^34^ seasonality^35^ and geographic area^34^. These asymptomatic infections are mostly submicroscopic (detected by molecular methods) and in some cases carry the transmission stages, gametocytes^36,37^. Half of the analyzed samples in our study cohort were positive for *P*. *falciparum* gametocytes showing a potential of high transmission intensity in the area. Similar to our finding, *P*. *falciparum* PCR-positive individuals had a 45% and 44% gametocyte carriage rate in a study conducted in Malawi^35^ and Kenya^36^, respectively. In several studies, gametocyte carriage was inversely associated with age of the infected person^38^; revealing that under-five year olds^39^ and school-age children^35^ were carrying more gametocytes than adults. We also observed that gametocyte carriage is more frequent in young children than in adults. Gametocyte density was similar in children and adults in our cohorts, though Coalson et. al reported a trend of lower gametocyte density in school-age children than other age groups^35^.

The chloroquine resistant genotype remains high in this *P*. *falciparum* parasite population. Similar results were found in previous studies showing that the dominant haplotype remains the resistant haplotype CVIET^9,40,41^. This is unexpected as CQ is no longer commonly available in the market for malaria treatment in the study area. However, the closely related drug (amodiaquine) that is given as *P*. *falciparum* first line treatment in combination with artesunate might play a role in selection of chloroquine resistant strains. While pfcrt CVIET is the most prevalent genotype in Africa, another resistance-associated genotype pfcrt SVMNT is rarely reported from Africa but more commonly found in South-America and some Asian countries^42^. The presence of SVMNT genotype was first reported in Tanzania in 2004 and later in Angola^43,44^. To our knowledge, this is the first time the presence of SVMNK genotype is detected in Gabon. SVMNT has been associated with the emergence of amodiaquine resistance^45^ and this finding highlights the need for continuous monitoring and re-evaluation of current therapy where amodiaquine, as in Gabon, is used in combination with artemisinin.

We did not evaluate the presence of CQ resistance in non-falciparum species in our study area where high level of *P*. *falciparum* resistant alleles is consistently reported after the withdrawal of the treatment. In general, except in the case of *P*. *vivax* infection, CQ-resistance is rarely reported in non-*falciparum* infection. Delayed parasite clearance and treatment failures of non-falciparum species are usually attributed to the special biology of these parasites (hypnozoites in case of *P*. *ovale* species, or longer life cycle, i.e. 72 hours in case of *P*. *malariae*), also considering stage specific activities of the drugs^46^. One study from Indonesia showed CQ resistance in *P*. *malariae* infections^47^, but this was not more repeatedly reported. As chloroquine resistance was never widely reported from other areas in non-falciparum species (except *P*. *vivax*), we would speculate that chloroquine remains efficacious against non-falciparum parasites in this area.

## Conclusion

Analysis by qPCR reveals the complexity of *Plasmodium* infection in this rural population in Gabon. For the final aim of elimination and eradication, also submicroscopic infections as well as non-falciparum species have to be considered to be able to sustain malaria control efforts. Otherwise, rural areas will always convey a reservoir for future infections.

## Methods

### Ethical approvals

Ethics approval was obtained from the responsible ethics committee Comité d´Ethique Institutionel of the Centre de Recherches Médicales de Lambaréné. Signed informed consent was obtained from adults ≥ 18 years or the legal guardian in case of minors, assent was additionally obtained from adolescents ≥ 12 years. All methods were performed in accordance with relevant guidelines and regulations.

### Study area and sampling

We performed a cross-sectional study in February/March 2016 to assess the prevalence of *Plasmodium* infections in 840 individuals aged from 1–96 years in a rural area of Gabon (Fougamou and villages in the surroundings). The area is characterized by close proximity to primary rain forest and small-scale farming. We invited everybody who lived in the chosen area and was older than 1 year to take part in the study. Blood was collected by venipuncture in an EDTA blood tube (Sarstedt), and temperature was taken by axillary measurement. A Giemsa-stained thin and a thick blood smear was performed and analyzed by Lambaréné method reading 100 microscopic fields on the same day by microscopy. All malaria cases (all species) that were diagnosed positive by thick blood smear were treated according to the national guidelines (three days treatment of artemether-lumefantrine or artesunate-amodiaquine irrespective of the Plasmodium species, no treatment for hypnozoites was given). Whole blood samples (500 µL) were collected mixed with 1,300 µL RNALater (Thermo Fisher Scientific) and stored at −20 °C until nucleic acid extraction was performed.

### Nucleic acids extraction

RNA*later* stabilized blood specimen were used for two types of extractions: either total nucleic acids (DNA and RNA) or total RNA automated in the QIAsymphony® SP system (Qiagen). Before total nucleic acids extraction with QIAsymphony DSP DNA kit, RNA*later* solution was removed by centrifugation and packed erythrocytes were resuspended with 1X PBS to a final volume of 420 μL and extracted by the QIAsymphony® SP as per the manufacturer’s instructions.

Total RNA extraction for *P*. *falciparum* gametocyte detection by RT-qPCR was done as reported earlier^48^ with some modifications. Briefly, frozen samples were thawed and half the volume of the blood-RNALater mix (900 µL) was separated, transferred to a 2-mL sample tube (Sarstedt, Numbrecht, Germany) and centrifuged for 3 min at 16,000 g. The supernatant was discarded and 300 µL buffer RTL Plus (Qiagen) supplemented with 1% (v/v) β-mercaptoethanol was added to the pellet and vortexed for 5 min before loading onto the QIAsymphony SP (Qiagen). RNA purification was automated using the QIAsymphony RNA Kit (Qiagen) according to the RNA CT 400 protocol provided with the instrument.

### Statistical analysis

All data was entered and reviewed using Excel, further analyses were done with GraphPad Prism version 5 and R v3.5.0. R packages tidyverse and funModeling were used for data processing and generation of graphics. Correlation of data was analyzed by Spearman test, data were compared by Mann Whitney U test, lines were fitted by linear regression. A p-value smaller than 0.05 was considered statistically significant.

### Ultra-sensitive RT-qPCR for parasite detection

Screening for *Plasmodium* infections was performed by pan-*Plasmodium* reverse transcription quantitative PCR (RT-qPCR) assay as described earlier^28^, with a lower limit of detection of 6 parasites/mL. The small subunit ribosomal RNA gene (18 S rDNA) as well as the expressed transcripts were co-amplified in a single step for high sensitivity detection of *Plasmodium* parasites. The amplicon was selected from a gene domain that is 100% conserved across human malaria species.

A result was interpreted as positive if the quantification threshold cycle of the amplification curve was below 40 (cutoff Cq < 40). The assay setup was performed in a sterile PCR workstation and the pipetting robot (QIAgility, Qiagen) was used for sample and mastermix dispensing into the wells of a 384 plate. The qPCR assay was carried out in the LightCycler 480 II (Roche Applied Science).

### Nested qPCR for species identification

Pan-*Plasmodium* assay positives were first subjected to conventional PCR amplification using primers by Snounou^49^ before further utilized as template for the qPCR. A limited cycle PCR was performed in a 50 µL reaction volume containing 7.5 µL of nucleic acids extract, 300 µM of each primer (PLU5 and PLU6), 1X Qiagen PCR buffer with 1.5 mM MgCl_2_, 250 µM dNTPs each, and 1U Taq DNA Polymerase (Qiagen). The cycling conditions were: Initial denaturation at 95 °C for 5 min, followed by 20 cycles of [95 °C for 30 s, 58 °C for 30 s, and 72 °C for 1:20 min] and a final extension at 72 °C for 5 min.

Single-plex qPCR assay was performed for five human malaria species (*P*. *falciparum*, *P*. *malariae*, *P*. *ovale curtisi*, *P*. *ovale wallikeri*, *and P*. *vivax*) using primers and probes described previously^28^. Briefly, each of the species-specific qPCR assays consisted of 2.5 µl of the pre-amplified product, 1X SensiMix II Probe No-ROX (Bioline), 300 µM of each primer pair and 150 µM of each probe (Supplementary Table S1) per 10 µL final reaction volume. Cycling conditions were: polymerase activation at 94 °C for 10 min, followed by 45 cycles of [95 °C for 10 s and 60 °C for 60 s]. Samples were tested in duplicate and all assays included a non-template control and positive control in duplicates. Quantification cycle values (Cq) were calculated by default using the second derivative maximum method integrated in the LightCycler 480 software (version 1.5.1.62). Positivity was considered after visual assessment of the amplification curves for variability between each sample replicates (standard deviation ≤ 1 cycle) and the quantification threshold value less than 40 (Cq < 40). Pan-*Plasmodium* PCR positive but species PCR negative samples were repeated by a standard PCR using primers specific for cytochrome B^50^ (n = 16) or 18 S rRNA genes^5^ (n = 6) and subsequent Sanger’s sequencing for samples with a high enough parasitemia (Cq below 36). All but one (*P*. *ovale*) of the sequencing results revealed a *P*. *falciparum* infection.

### Generation of standard curve for quantification of parasite load by PCR

A standard curve of highly synchronized ring-stage *P*. *falciparum* 3D7 laboratory strain was prepared by using the extracted total nucleic acid to estimate parasitemia. Quantification of parasites was done by extrapolating Cq values to the standard curve based on linear regression analysis. Therefore, quantification is not fully equivalent to microscopic reading as in clinical infections in addition to ring stage parasites, gametocytes as well as later asexual stages might be present, especially in non-falciparum species infections. For the species-specific PCR, quantification is only approximate as evaluation was done after preamplification, which allows at most ordinal comparison between infections.

### Detection of *Plasmodium falciparum* gametocytes in blood by RT-qPCR

Among samples positive for *P*. *falciparum* by qPCR, a subset (n = 223) of samples was selected and analyzed by RT-qPCR for the presence of gametocytes in the infected participants. Gametocyte specific qPCR was performed to measure the transcript levels of *Pfs25* mRNA using previously published targets, with modifications. A Taqman RNA-to-CT™ 1-Step kit (Thermo Fisher Scientific) was used for the quantitation of the gene expression levels. The reaction contains 1 × Taqman enzyme mix, 150 nM probe, 1 × Taqman RTqPCR mix, 400 nM of each primer and 2.5 µL of RNA extract.

The reaction conditions consisted of reverse transcription (48 °C/20 min), enzyme activation (96 °C/10 min), and two-temperature cycling steps (45 cycles, 95 °C for 15 s and 62 °C for 1 min). The Cq value was determined as above for the species qPCR. All samples were tested in triplicates together with the non-RT and non-template controls. Samples were considered positive when a Cq value below 40 was present in at least two technical replicates. The used primer and probe sequences were published previously^48^ and are given in S1. Gametocyte carriage was only evaluated for *P*. *falciparum* and not for the other Plasmodium species.

### *P*. *falciparum* gametocyte detection by microscopy

The samples positive by qPCR for Pfs25 were re-read by microscopy to check for *P*. *falciparum* gametocytes. 300 microscopic fields were read using the Lambaréné method by two independent experienced microscopists.

### PfCRT genotyping

A multiplex qPCR assay was used to genotype Pfcrt gene spanning codons 72–76 using previously described hydrolysis probes and primers with modifications^51,52^. To enhance sensitivity, a preamplification followed by qPCR was done with primers listed in S1. In brief, a conventional PCR was carried out using PfCRT_Preamp1 & PfCRT_Preamp2 for 20 cycles in 25 μL reaction volume. Limited PCR-cycled products were used as templates in a qPCR multiplex reaction containing three hydrolysis probes. Each probe representing one of the three Pfcrt haplotypes; the CQ sensitive haplotype (CVMNK), and the two CQ resistance-associated haplotypes (CVIET, SVMNT). Different fluorophores were tagged to each probe enabling detection of the haplotypes in parallel from each sample. DNA extracted from *P*. *falciparum* strains 3D7, Dd2, and 7G8 were used as positive controls for haplotypes CVMNK, CVIET and SVMNT, respectively.

## Supplementary information

Supplement

## Data Availability

The datasets used and/or analyzed during the current study are available from the corresponding author on reasonable request.
