# Supplementary material for: Characterization of Plasmodium infections among inhabitants of rural areas in Gabon
Source: Sci Rep. 2019 Jul 5;9:9784. doi: 10.1038/s41598-019-46194-9 (PMC6611864; doi:10.1038/s41598-019-46194-9)
Supplement: Supplementary file 1 — Supplement [file 41598_2019_46194_MOESM1_ESM.docx]

**Supplementary Information:**

**Title: Characterization of Plasmodium infections among inhabitants of rural areas in Gabon**

**Running title: *Plasmodium* species complexity**

Tamirat Gebru Woldearegai^1,2,3#^, Albert Lalremruata^1,2,3#^, The Trong Nguyen^1,2,3,4^, Markus Gmeiner^1,2,3^, Luzia Veletzky^3,6^, Gildas B Tazemda-Kuitsouc^5^, Pierre Blaise Matsiegui^3,5^, Benjamin Mordmüller^1,2,3^, Jana Held^1,2,3^*

**Supplementary Table 1: Primer and probe sequences for differential diagnosis of *Plasmodium* species**

| Primer and probe name | Sequence (5'-3') | 5' Modification | | 3' Modification | Genus/species | Reference |
| --- | --- | --- | --- | --- | --- | --- |
| rPLU6 | TTAAAATTGTTGCAGTTAAAACG |  |  | | *Plasmodium* | Snounou *et.al*.^1^ |
| rPLU5 | CCTGTTGTTGCCTTAAACTTC |  |  | |  |  |
| PLU | GCTCTTTCTTGATTTCTTGGATG |  |  | | *Plasmodium* | Mordmüller *et.al*.^2^ |
| PLU | AGCAGGTTAAGATCTCGTTCG |  |  | |  |  |
| PLU-Probe | ATGGCCGTTTTTAGTTCGTG | HEX | MGBEQ | |  |  |
| Pf-1 | ATTGCTTTTGAGAGGTTTTGTTACTTT |  |  | | *P. falciparum* | Veron *et.al*.^3^ |
| Pf-2 | GCTGTAGTATTCAAACACAATGAACTCAA |  |  | |  |  |
| Pf-Probe | CATAACAGACGGGTAGTCAT | HEX | MGBEQ | |  |  |
| Pm-TFwd | GGTGTTGGATGATAGAGTAA |  |  | | *P. malariae* | Groger *et.al*.^4^ |
| Pm-TRev | CCCAAAGACTTTGATTTCTC |  |  | |  |  |
| Pm-Tprobe | AGGAAGCTATCTAAAAGAAACACTCAT | HEX | BHQ-1 | |  |  |
| PO-18S-S-Fwd | ATTTCAAAGAGTCATGGCGTTTCTG |  |  | | *P. ovale curtisi* | Groger *et.al*.^4^ |
| POC-18S-S-Rev | TTGTAAAGGAGACACTTTCTTGAAATCG |  |  | |  |  |
| POC-18S-S-Probe | CTCCTTGGTCGATCTGCCCAGCACT | FAM | BHQ-1 | |  |  |
| PO-18S-S-Fwd | ATTTCAAAGAGTCATGGCGTTTCTG |  |  | | *P. ovale wallikeri* | Groger *et.al*.^4^ |
| POW-18S-S-Rev | TGTAAAGGAGACAACTTTCTTGGAGCTA |  |  | |  |  |
| POW-18S-S-Probe | TTGATCGCCCAGCACTGACCATCT | HEX | BHQ-1 | |  |  |
| VIV-F | GCAACGCTTCTAGCTTAATCCAC |  |  | | *P. vivax* | Kamau *et.al*.^5^ |
| VIV-R | CAAGCCGAAGCAAAGAAAGTCC |  |  | |  |  |
| VIV-Probe | ACTTTGTGCGCATTTTGCTA | HEX | MGBEQ | |  |  |

HEX 6-hexachlorofluorescein. FAM 6-carboxyfluorescein. MGBEQ minor groove binder eclipse quencher. BHQ-1 black hole quencher-1. rRNA ribosomal ribonucleic acid.

**Primer and probe sequences for the detection of *P. falciparum* gametocytes**

| Primer and probe name | Sequence (5'-3') | 5' Modification | 3' Modification | Genus/species | Reference |
| --- | --- | --- | --- | --- | --- |
| Pfs25_Fwd | GACTGTAAATAAACCATGTGGAGA |  |  | *P. falciparum (gametocytes)* | Gebru *et.al*.^6^ |
| Pfs25_Rev | CATTTACCGTTACCACAAGTTA | LC640 | BHQ-2 |  |  |
| Pfs25_Probe | AGATGGAAATCCCGTTTCATACGCTTGT |  |  |  |  |

**Primer and probe sequences for genotyping PfCRT by real-time PCR**

| Primer and probe name | Sequence (5'-3') | 5' Modification | 3' Modification | Reference |
| --- | --- | --- | --- | --- |
| PfCRT_Preamp1 | TGGCTCACGTTTAGGTGGAGGTTCTTG |  |  | Nag *et.al.^7^* |
| PfCRT_Preamp2 | ACTGAACAGGCATCTAACATGGATATAGC |  |  |  |
| Pfcrt_Fwd | TGGTAAATGTGCTCATGTGTTT |  |  | Sutherland *et.al.^8^* |
| Pfcrt_Rev | AGTTTCGGATGTTACAAAACTATAGT |  |  |  |
| CVMNK_probe | TGTGTAATGAATAAAATTTTTGCTAA | FAM | BHQ1 |  |
| CVIET_probe | TGTGTAATTGAAACAATTTTTGCTAA | HEX | BHQ1 |  |
| SVMNT_probe | AGTGTAATGAATACAATTTTTGCTAA | CY5 | BHQ1 |  |

Underlined and highlighted bases correspond to mutant bases.

References:

^1^ Snounou G, Viriyakosol S, Zhu XP, Jarra W, Pinheiro L, do Rosario VE, et al. High sensitivity of detection of human malaria parasites by the use of nested polymerase chain reaction. Molecular and biochemical parasitology. 1993 Oct;61(2):315-20. PubMed PMID: 8264734

^2^ Mordmüller B, Surat G, Lagler H, Chakravarty S, Ishizuka AS, Lalremruata A, et al. Sterile protection against human malaria by chemoattenuated PfSPZ vaccine. Nature. 2017;542:445–9. PubMed PMID: 28199305

^3^ Veron V, Legrand E, Yrinesi J, Volney B, Simon S, Carme B. Genetic diversity of msp3alpha and msp1_b5 markers of Plasmodium vivax in French Guiana. Malaria journal. 2009 Mar 11;8:40. PubMed PMID: 19284592. Pubmed Central PMCID: 2660359.

^4^  Groger M, Veletzky L, Lalremruata A, Cattaneo C, Mischlinger J, Zoleko-Manego R, et al. Prospective Clinical Trial Assessing Species-Specific Efficacy of Artemether-Lumefantrine for the Treatment of Plasmodium malariae, Plasmodium ovale, and Mixed Plasmodium Malaria in Gabon. Antimicrob Agents Chemother. 2018;62. PubMed PMID: 29311086.

^5^ Kamau E, Alemayehu S, Feghali KC, Saunders D, Ockenhouse CF. Multiplex qPCR for detection and absolute quantification of malaria. PloS one. 2013;8(8):e71539. PubMed PMID: 24009663.

# ^6^ [Gebru T](https://www.ncbi.nlm.nih.gov/pubmed/?term=Gebru%20T%5BAuthor%5D&cauthor=true&cauthor_uid=28800735), [Lalremruata A](https://www.ncbi.nlm.nih.gov/pubmed/?term=Lalremruata%20A%5BAuthor%5D&cauthor=true&cauthor_uid=28800735), [Kremsner PG](https://www.ncbi.nlm.nih.gov/pubmed/?term=Kremsner%20PG%5BAuthor%5D&cauthor=true&cauthor_uid=28800735), [Mordmüller B](https://www.ncbi.nlm.nih.gov/pubmed/?term=Mordm%C3%BCller%20B%5BAuthor%5D&cauthor=true&cauthor_uid=28800735), [Held J](https://www.ncbi.nlm.nih.gov/pubmed/?term=Held%20J%5BAuthor%5D&cauthor=true&cauthor_uid=28800735). Life-span of in vitro differentiated Plasmodium falciparum gametocytes. [Malar J.](https://www.ncbi.nlm.nih.gov/pubmed/?term=Life-span+of+in+vitro+differentiated+Plasmodium+falciparum+gametocytes) 2017;11;16(1):330.

^7^ Nag S, Dalgaard MD, Kofoed P-E, Ursing J, Crespo M, Andersen LO, et al. High throughput resistance profiling of Plasmodium falciparum infections based on custom dual indexing and Illumina next generation sequencing-technology. Sci Rep. 2017;7:2398.PubMed PMID: 28546554

^8^ Sutherland CJ, Haustein T, Gadalla N, Armstrong M, Doherty JF, Chiodini PL. Chloroquine-resistant Plasmodium falciparum infections among UK travellers returning with malaria after chloroquine prophylaxis. J Antimicrob Chemother. 2007;59:1197–9. PubMed PMID: 17475629
